# Relationship of actigraphy-assessed sleep efficiency and sleep duration to reactivity to stress

**DOI:** 10.5935/1984-0063.20190090

**Published:** 2019

**Authors:** Matthew N. Eiman, Julia Mary Louise Pomeroy, Ali A. Weinstein

**Affiliations:** 1 George Mason University, Center for the Study of Chronic Illness and Disability - Fairfax - Virginia - United States.

**Keywords:** Sleep Deprivation, Blood Pressure, Heart Rate, Cardiovascular Disease, Stress, Physiological

## Abstract

**Objective:**

Sleep duration is an important predictor of cardiovascular health outcomes, but the role of sleep efficiency is less clear. This study investigated actigraphy-assessed sleep efficiency and sleep duration and their relationship with responses to mental and physical challenge tasks.

**Methods:**

To record sleep, actigraph devices were worn on the wrist continuously by 25 participants (age: 33.9±6.9, 60% female) for the duration of a seven-day period. Movement data were used to estimate sleep duration and efficiency. Mental (Stroop test) and physical (cold pressor) challenges were used to assess reactivity to and recovery from stress. During these tasks, heart rate, blood pressure, and emotional states were measured.

**Results:**

Significant findings from the mental challenge included a negative correlation between sleep efficiency and reaction time. There were no significant relationships between sleep efficiency and cardiovascular measures during the mental challenge, but sleep duration was related to cardiovascular reactivity. For the physical challenge, sleep efficiency was positively and significantly correlated with blood pressure recovery and sleep duration was not related to any outcome measures.

**Discussion:**

Previous literature has focused on sleep duration when assessing sleep and cardiovascular outcomes. However, sleep efficiency may be equally or more important when investigating reactivity to and recovery from stress.

## INTRODUCTION

Sleep is widely recognized as an important indicator of health. It is currently recommended that adults between the ages of 19 and 64 sleep about 7-9 hours a night^1^. A large body of literature documents that duration of sleep is an important predictor of physical and mental health problems. Evidence from three longitudinal, prospective, population-based studies has demonstrated that progressively shorter (≤ 6 hours per night) or longer (≥ 9 hours per night) sleep duration is associated with all-cause age-specific mortality, including a 15% increase in risk for mortality among individuals getting less than five hours a night^2^^-^5. Suboptimal sleep duration is also associated with specific health issues including increased risk for weight gain, obesity and type II diabetes^6^^-^10, depression, alcohol abuse, and impairments in cognitive functioning^11^^,^^12^, among others. Sleep duration has important consequences for myriad issues and health overall.

One increasing area of concern has been the connection between sleep duration and poor cardiovascular outcomes. As compared to normal sleep duration (population mean is about 7-8 hours per night), shorter or longer sleep duration is associated with cardiovascular morbidity and mortality including that from hypertension, coronary heart disease, heart failure, myocardial infarction, and stroke^2^^,^^8^^,^^13^^-^18.

Compared to sleep quantity, however, there has been less investigation into the impact of sleep quality on cardiovascular outcomes. Insomnia (difficulty falling and/or staying asleep) is the most commonly reported sleep problem and thus the primary focus of most sleep quality studies^2^^,^^19^. Kabat et al.^13^ found that insomnia, unlike total sleep duration, was not related to mortality from cardiovascular disease in a sample of more than 158,000 women. These authors, however, used self-reported, secondary data to assess the relationship. Sleep efficiency - a measure of quality of sleep, defined as the ratio of total time spent asleep compared to total time in bed - has been less studied. Castro-Diehl et al.^20^ found that both short sleep duration and lower sleep efficiency were related to lower high-frequency heart rate (HR) variability, which has been associated with an increased risk of cardiovascular disease. Reinhard et al.^21^ demonstrated that sleep efficiency, unlike sleep duration, was more predictive of mortality in a sample of chronic heart failure patients.

Despite mixed evidence regarding the connection between sleep quality and cardiovascular outcomes, this small body of research suggests that, while sleep duration has been the more widely used measure of sleep, sleep efficiency may also have valuable predictive power. Sleep efficiency may more precisely measure sleep deficiencies stemming from internal cognitive or biobehavioral processes than does sleep duration, which can be restricted by daily routines^22^. For example, suboptimal sleep duration can be secondary to social jetlag and other external issues such as personal obligations, shift work, or lifestyle factors that cut sleep short^23^^-^25 but are not reflective of endogenous sleep deficiencies. Accordingly, it is important that sleep efficiency, both as a measure of sleep and as a potential predictor of cardiovascular outcomes, is better understood.

Moreover, little is understood regarding the pathways through which sleep deficiencies translate into cardiovascular morbidities. Some researchers suggest that the relationship is not directly causal, but that it is mediated by other factors^13^^,^^26^. Two potential mediators proposed to date are increases in blood pressure and sympathetic hyperactivity^2^. Increases in blood pressure, HR, and sympathetic activity have been shown to potentiate increased myocardial oxygen demand that coincides with morning cardiovascular events^26^^-^29. Kato et al.^26^ hypothesized that sleep deprivation would increase blood pressure, HR, and sympathetic activity in a sample of healthy individuals, and that these cardiovascular measures would fluctuate more under exposure to stressful stimuli. They found that resting blood pressure was higher in sleep-deprived individuals as compared to their rested counterparts, supporting the idea that blood pressure could be a mechanism of action. In contrast, however, heightened sympathetic activity was not observed, and there was no between-group variation in physiological response to the stressful stimuli. Nonetheless, their sample included only eight individuals who were observed for a short period (two nights)^26^.

In addition to blood pressure, the possibility that sympathetic hyperactivity to stressors could serve as a link between sleep efficiency and cardiovascular morbidity presents a fascinating, albeit understudied prospect. Similar to the link between sleep loss and cardiovascular morbidity, there are known associations between sleep loss and reactivity to stress. For example, insomnia is often described by its sufferers as a symptom of hyperarousal^2^^,^^30^, and elevated stress levels are thought to play a key role in potentiating sleeplessness. While stress may result in poor sleep, poor sleep can also induce stress^31^^,^^32^. It is conceivable, then, that individuals with poor sleep efficiency might be more reactive to stressful stimuli.

In turn, physiological reactivity to and recovery from stress are important indicators of cardiovascular health. Two meta-analyses have reported positive associations between cardiovascular reactions to acute stress and future blood pressure status^33^^,^^34^. Carroll et al.^33^ also reported relationships between cardiovascular reactivity and coronary atherosclerosis, cardiovascular disease morbidity, and mortality. Furthermore, they found lower physiological reactivity to stress was associated with depression and obesity^33^.

Chida & Steptoe^34^ found that lower physiological reactivity to stress was related to development of cardiovascular disease. A comprehensive meta-analysis by Panaite et al.^35^ found that poor cardiovascular recovery from both physical and psychological challenges was related to poor cardiovascular outcomes, with the effects being stronger for physical challenges as compared to mental ones. As with sleep, research regarding physiological reactivity to stress suggests that over-reactivity (as with over-sleeping) and under-reactivity (as with under-sleeping) are related to deleterious cardiovascular outcomes.

Considering that high stress levels can potentiate poor cardiovascular outcomes, sleep inefficient individuals, if more stressed, may experience poorer cardiovascular outcomes. Previous research shows that poor sleep efficiency results in higher blood pressure reactivity to psychosocial stress^36^. In another study, young adults that were sleep deprived had higher systolic blood pressure (SBP) reactivity to psychosocial stress^37^. In a naturalistic study, Mezick et al.^38^ found that shorter duration of actigraphy-assessed sleep in young adults was associated with poorer HR and diastolic blood pressure (DBP) recovery after cognitive stress.

In a college-aged sample assessed in a non-laboratory setting, Bassett et al.^39^ found that higher self-reported sleep quality, not sleep duration, was related to abnormally high cortisol responses to stress, while lower self-reported sleep efficiency was associated with blunted cortisol responses. Consequently, it appears that reactivity to and recovery from stress may represent an important potential pathway between poor sleep efficiency and cardiovascular health. However, mechanisms of action between sleep and cardiovascular outcomes remain unclear. Given the high prevalence of sleep disorders in society and the lack of research examining relationships between sleep quality, stress response, and cardiovascular outcomes, this is an important area of study^2^.

The aim of the current investigation was to investigate sleep efficiency, as assessed by ambulatory monitoring, and its relation to physiological reactivity to and recovery from stress. We hypothesized that less efficient sleep and shorter sleep duration would be associated with increased physiological reactivity to and recovery from stress, as measured through SBP, DBP, and HR.

## METHODS

### Participants

The individuals included in this analysis were part of a larger trial investigating the effects of exercise withdrawal^40^ and therefore had to be “regularly exercising,” defined as engagement in aerobic exercise for a continuous 30 minutes at least three days a week for the past six months. Participants were recruited through newspaper and local advertisements, then screened via phone using the following exclusion criteria: 1) age less than 18 or more than 45 years old; 2) current and regular use of anti-inflammatory or anti-coagulation medication other than aspirin; 3) history of cardiovascular disease; 4) current hypertension diagnosis (i.e., blood pressure greater than 140/90 mmHg); 5) obese (body mass index greater than 30 kg/m^2^); and, 6) current treatment for a psychiatric or psychological disorder. Participation lasted one week and included cardiovascular monitoring, psychological assessments, and psychological questionnaires administered during lab visits at baseline and a one-week follow-up visit. All testing was completed in the morning between 7 and 9 am to control for the possible effect of time of day on cardiovascular measurements. The study was approved by the Institutional Review Board of the Uniformed Services University of the Health Sciences and each participant provided written informed consent.

### Activity Monitoring

An actigraph accelerometer wristband (Actiwatch, Mini-Mitter Co, Bend, OR) was used to record the activity of each participant. Participants were instructed to wear it continuously throughout the day and night for seven consecutive nights (including weekends). The Actiwatch recorded all movement over five-minute epochs and calculated periods of sleep that resulted in parameters of sleep duration, time spent asleep and awake, and sleep efficiency. Sleep efficiency was conceptualized as the percentage of total time in bed actually spent asleep and was operationalized as time asleep divided by the number of minutes in the rest interval. The Actiware software automatically detects the rest interval and the number of minutes asleep during that time. Actigraphy and the software-derived algorithm has been validated as an alternative to polysomnography in measuring sleep parameters^41^.

To be included in the analysis, participants had to register at least four valid nights of sleep in the weeklong (seven-night) time period. This requirement eliminated 15 of the 40 individuals who participated in the larger trial from inclusion in the present analysis, resulting in a final sample of 25 individuals. Actigraphy readouts were examined to identify and exclude nights for which participants had taken off the accelerometer (e.g., a sleep period where not even minimal activity was present). Additionally, sleep periods of greater than 720 minutes (12 hours) and less than 180 minutes (3 hours) were excluded from analysis. Sleep duration and sleep efficiency scores were averaged across days for analysis. Participants varied on the number of weekday and weekend nights that were included, but all participants had more weekday nights included than weekend nights. These values ranged from 0% to 40% weekend nights included, with the average being 25% of included nights being weekend nights. Weekend nights were defined as Friday night to Saturday morning and Saturday night to Sunday morning.

### Cardiovascular Measurements

After placement of an automated blood pressure monitor (Critikon Ditimap), a 30-minute baseline (rest) period was completed in order to establish baseline hemodynamic measures (SBP, DBP, and HR). During the last ten minutes of the resting period, the hemodynamic measures were obtained at 2-minute intervals. Baseline hemodynamics were determined by averaging the last three resting measures during the rest period. During the mental and physical challenge tests, the hemodynamics were also assessed every two minutes, starting with a measurement at 30 seconds into the test and then following at 2 minutes and 30 seconds into the test. To assess recovery, after each of the challenge tests, the same measures (SBP, DBP, and HR) were assessed every two minutes. A recovery factor for each challenge test was calculated by subtracting the last measurement during the test from the first measurement after the test.

### Mental Challenge Test

A Stroop test was used to measure each participant’s response to a mental challenge task. In this test, participants sat in front of a computer screen. The top half of the screen displayed the words “red”, “green”, or “blue”, however each word could be written in a different color than that denoted by the word. For instance, the word “blue” might be written in red ink. The bottom half of the screen presented the words “red”, “green”, and “blue”. Participants had to match the color of the word displayed on the top half of the screen with the actual word for that color on the bottom half of the screen. For example, if the top half of the screen presented the word “blue” but was written in the color red, the participant had to select the word “red” in the bottom half of the screen. As participants answered correctly, the test increased in difficulty. If a selection was not made after a couple of seconds, the words disappeared and the answer was recorded as “no response”.

### Physical Challenge Test

A cold pressor test was used to measure each participant’s response to a physical challenge task. This test required participants to place their dominant hand in ice water for a maximum of 180 seconds. Equal parts ice and water were added into the mixture. Not every participant made it to the first measurement of hemodynamic response (30 seconds into the challenge), therefore the sample size for recovery factor measurements was 22. The cold pressor test has been validated as a method to elicit cardiovascular reaction and can predict subsequent development of hypertension^42^.

### Psychological Measures

Individual Likert-type rating scales were used to measure emotional states before and after the challenge tests. Measures were taken before and after the Stroop and cold pressor tests. Each response was rated on a scale from 1 (not at all) to 7 (very much). The emotional states assessed were: anxious, frustrated, irritated, tired, challenged, amused, stressed, depressed, interested, angry, involved, happy, and pain. An emotional disturbance score was created by summing the negative emotions (anxious, frustrated, irritated, tired, stressed, depressed, angry, and pain) and subtracting their total from the positive emotions (amused and happy). These Likert evaluations were derived from the Profile of Mood States (POMS)^43^. The full POMS is a 64-item long evaluation and the participants were asked to fill-out the items four times (before and after each of the challenge tests), therefore to reduce participant burden, we selected the most relevant items from the POMS for this investigation.

### Statistical Analyses

Pearson correlations were used to assess relationships between variables. Statistically significant correlations were then investigated further with linear regression to determine the relative predictive power of sleep efficiency and sleep duration. For each test, a repeated measures ANOVA was employed to compare change over time between the baseline, peak, and follow-up measurements. Post-hoc analyses included paired sample t-tests, which were used to investigate the changes between time points as revealed in the ANOVA. Analyses were performed using SPSS 24 (IBM SPSS Statistics for Macintosh, Version 24.0.0.0)^44^. *P*-values less than .05 were considered statistically significant. Data are presented below as means followed by their standard deviations.

## RESULTS

The final sample size included 25 individuals (except when noted). Participants had an average age of 33.9 years (*SD*=6.9) and were about 60% female and 80% white. Additional demographic characteristics are presented in Table 1.

**Table 1:** Participant Characteristics.

Characteristics (N=25)	
Age, mean (SD), years	33.9 (6.9)
Female, No. (%)	15 (60.0)
Race/ethnicity, No. (%)*	
Caucasian, not Hispanic or Latino	20 (80.0)
Black or African-American	4 (16.0)
Asian	1 (4.0)
Body mass index, mean (SD)	22.8 (2.6)
Sleep Duration, mean (SD), minutes	456.5 (50.7)
Sleep Efficiency, mean (SD), percent	83.5 (5.0)

* None of the participants identified as Hispanic or Latino, American Indian/Alaska Native, or Native Hawaiian/Pacific Islander.

### Mental Challenge (Stroop Test)

#### Cardiovascular Outcomes

Cardiovascular outcomes for the Stroop test are described in Table 2 and recovery factor measurements are in Table 3. All three cardiovascular measurements SBP (*F*(2,24)=27.13, *p*<.001), DBP (*F*(2,24)=71.91, *p*<.001), and HR (*F*(2,24)=37.54, *p*<.001) showed significant changes between resting and peak and peak and recovery measurements. Furthermore, no significant differences were noted between resting and recovery measurements.

**Table 2:** Cardiovascular Outcomes.

Point of Measurement	Systolic Blood Pressure	Diastolic Blood Pressure	Heart Rate
Resting (Baseline)	109.7 (8.2)	68.1 (7.5)	65.3 (9.9)
Stroop Test			
Peak	120.0 (13.1)	77.4 (8.9)	77.2 (13.2)
Last Measure during Stroop	117.0 (13.9)	74.4 (8.9)	73.4 (11.3)
First Recovery after Stroop	107.9 (11.7)	69.1 (8.8)	67.2 (9.5)
Cold Pressor Test			
Peak	137.8 (23.8)	89.6 (10.5)	75.8 (7.9)
First Measure during Cold Pressor	128.5 (22.9)	85.3 (12.0)	73.5 (9.4)
First Recovery after Cold Pressor	111.4 (13.9)	69.6 (8.7)	63.5 (11.0)

Numeric values are expressed as mean (SD).

**Table 3:** Pearson Correlations between Sleep Duration/Efficiency and Cardiovascular/Psychological Outcomes.

Measure	Stroop Test		Cold Pressor Test	
	Sleep Duration	Sleep Efficiency	Sleep Duration	Sleep Efficiency
Cardiovascular Outcomes				
Difference in Resting and Peak SBP	0.235	0.105	0.235	0.105
Difference in Resting and Peak DBP	0.212	-0.109	0.212	-0.109
Difference in Resting and Peak HR	.490*	0.256	0.140	0.117
Recovery Factor SBP	0.281	-0.147	0.286	.465*
Recovery Factor DBP	.429*	0.368	0.233	.511*
Recovery Factor HR	0.233	-0.1	0.002	0.127
Psychological Outcomes				
Baseline, Emotional Disturbance	-0.087	0.319	-0.151	-0.042
Performance on Challenge Tasks				
Stroop Test - Reaction Time	-0.127	-.537*	*n/a*	*n/a*
Stroop Test - Correct Answers	0.036	0.420	*n/a*	*n/a*
Cold Pressor Test - Time Endured	*n/a*	*n/a*	0.333	0.248

* Significant at the 0.05 probability level.

Abbreviations: SBP, systolic blood pressure; DBP, diastolic blood pressure; HR, heart rate

#### Psychological Outcomes

The average baseline emotional disturbance score was 5.4 (*SD*=3.6) and the average Stroop emotional disturbance score was 11.5 (*SD*=6.6). ANOVAs revealed that the average baseline and post-Stroop emotional disturbance scores were statistically different from one another (*F*(1, 23)=27.70, *p*<.001).

#### Sleep Outcomes and Stroop Test Performance

Pearson correlation coefficients that describe the associations between sleep duration, sleep efficiency, cardiovascular outcomes, and psychological outcomes are presented in Table 3. The mean reaction time for correct answers among participants was 1.3 seconds (*SD*=.43) and the average number of correct answers was 95.4 (*SD*=33.6). The average number of incorrect responses and “no answer” responses were 17.6 (*SD*=12.3) and 32.6 (*SD*=14.5), respectively. With the Stroop test performance metrics, sleep efficiency negatively and significantly correlated with reaction time to correct responses, *r*(24)=-.54, *p*=.006, and strongly, though insignificantly correlated with number of correct answers (*r*(24)=.420). Sleep duration did not significantly correlate with either of these performance metrics. For reaction time to correct responses, sleep duration and sleep efficiency explained 29.1% (27.9% explained by sleep efficiency) of the variance. With regard to psychological outcomes, sleep efficiency significantly correlated with the change between the baseline and follow-up “challenged” scores *r*(24)=.44, *p*=.026, and strongly correlated with the change between baseline and follow-up emotional disturbance scores (*r*(24)=.319). Sleep duration did not show any significant or strong correlations with the psychological measures. Regarding cardiovascular outcomes, a moderate correlation existed between sleep efficiency and the difference in resting and peak HR (*r*(24)=.256) and the DBP recovery factor (*r*(24)=.368). Sleep duration yielded two strong and significant correlations with the DBP recovery factor (*r*(24)=.42, *p*=.0325) and the difference between resting and peak HR (*r*(24)=.49, *p*=.0129). For these relationships, sleep duration and sleep efficiency explained 24.4% (12.6% explained by sleep duration) of the variance in the DBP recovery factor and 25.2% (19.9% explained by sleep duration) of the variance in the difference between resting and peak HR.

### Physical Challenge (Cold Pressor Test)

#### Cardiovascular Outcomes

Cardiovascular outcomes for the cold pressor test are described in Table 2 and recovery factor measurements are in Table 3. ANOVAs revealed statistically significant changes between baseline, peak, and post-cold pressor scores in SBP (*F*(2,21)=42.90, *p*<.001), DBP (*F*(2,21)=97.03, *p*<.001), and HR (*F*(2,21)=31.55, *p*<.001) measurements. ANOVA analysis also showed no statistical significance between resting and recovery measurements.

#### Psychological Outcomes

As with the mental challenge, changes in emotions were observed throughout the physical challenge. On average, the emotional disturbance score after the cold pressor test was 14.8 (*SD*=8.0). This differed from the average baseline measurement by a mean of 9.5 points (*SD*=8.2). The change between the baseline and cold pressor emotional disturbance scores was significantly different (*t*(24)=-5.77, *p*<.001)*.*

#### Sleep Outcomes and Cold Pressor Test Performance

Participants averaged 129.4 seconds (*SD*=64.4) in the cold pressor test. Sleep efficiency and sleep duration were moderately correlated with average time endured (*r*=.24 and .33, respectively), though these findings were not significant. Neither sleep outcome exhibited a significant correlation with the emotional measures. With respect to cardiovascular outcomes, however, sleep efficiency strongly and significantly correlated with the SBP recovery factor with a coefficient of *r*=.47 (*p*=.029) and the DBP recovery factor with a coefficient of *r*=.51 (*p*=.015). For these relationships, sleep duration and sleep efficiency explained 24.9% (18.2% explained by sleep efficiency) of the variance in the SBP recovery factor and 27.5% (23.3% explained by sleep efficiency) of the variance in the DBP recovery factor.

## DISCUSSION

This study explored the relationship between sleep efficiency, sleep duration, and responsiveness to mental and physical challenges. The vast majority of published research in this area has focused on sleep duration, whereas sleep efficiency has been largely ignored. However, sleep efficiency may be an important predictor of cardiovascular health as it distinguishes between the total time spent in bed and total time asleep. As highlighted in this study, sleep efficiency was more related to cardiovascular recovery after a physical challenge than sleep duration. Poor cardiovascular recovery after laboratory challenges predicts adverse cardiovascular outcomes^35^. Accordingly, a better understanding of cardiovascular reactivity and recovery from stress may help to elucidate the connection between sleep quality and cardiovascular health. Notably, prior research has not indicated whether measures of sleep efficiency might be comparable, or even superior, to sleep duration in predicting various cardiovascular outcomes. For these reasons, sleep efficiency as a predictor of health warrants further exploration.

In both the Stroop and cold pressor test, cardiovascular measurements changed significantly from pre-test to mid-test and mid-test to post-test. This indicates both tests succeeded in eliciting physiological responses from the participants. Furthermore, participants felt more emotionally disturbed from baseline to post-test measurements in both challenge tests. These emotional changes show that participants were actively engaged in the two challenge tests.

Additionally, higher sleep efficiency correlated with faster average reaction time for correct responses on the Stroop test. Although not statistically significant, there was a strong correlation between higher sleep efficiency and more correct responses on the Stroop test (*r*=.420). These data indicate that participants with higher sleep efficiencies could respond quicker and more accurately than their sleep inefficient counterparts and may demonstrate better cognitive performance.

There were strong correlations between sleep efficiency and both the SBP and DBP recovery factors during the cold pressor test. Higher sleep efficiency correlated with a larger recovery of blood pressure after the physical challenge. Previous research has linked high cardiovascular reactions to a cold pressor as a predictor of hypertension^45^. Furthermore, a meta-analysis by Chida & Steptoe^34^ found evidence that sustained cardiovascular reactions or slow recovery after mental stress predict poor future cardiovascular status. Given that cardiovascular reactions in the cold pressor test varied by sleep efficiency, we suggest that sleep efficiency may affect how well one’s heart can recover from stress, with implications for future cardiovascular health. This conclusion would be consistent with and additive to previous research that has linked poor sleep duration and hypertension, among other cardiovascular morbidities^11^^,^^14^^,^^15^.

The current study demonstrated the predictive power of sleep efficiency compared to sleep duration in certain scenarios including both cognitive performance (reaction time) and cardiovascular recovery from a physical challenge. Sleep duration showed statistical significance with the change in resting to peak HR and with the DBP recovery factor on the Stroop test. These findings suggest that sleep efficiency warrants further investigation into the role it plays in cardiovascular health outcomes and cognitive performance.

The primary limitation of this study was the small sample size, which may have impacted the statistical power of the analyses. The current study was a secondary analysis of a larger trial that was an experimental trial of exercise withdrawal. Therefore, the sample size calculation for the study was based on the primary outcome of the experimental trial and not the analyses of sleep. Even with the small sample size for the sleep analysis, statistically significant correlations were found. In all of the presentation of the results, all correlations (statistically significant and not statistically significant) are presented so that the magnitude of relationship (which is independent of sample size) can be seen.

Additionally, not all participants made it to the 30-second time point to have their blood pressure measured during the cold pressor test, which reduced the sample size for those analyses. Nonetheless, we consider the sample size to be an improvement relative to that of other studies using actigraphy over an extended period of time^26^. We also believe that the limited sample is acceptable given the exploratory nature of this study in investigating sleep efficiency with ambulatory monitoring.

It is also worth noting that this sample was composed of regularly exercising adults, since the larger investigation was of an experimental exercise withdrawal trial. Exercise is known to improve sleep outcomes^46^ and cardiovascular health^47^; therefore, this sample likely had better than average sleep and cardiovascular outcomes, and results may represent a conservative estimate. Using this sample allowed us to estimate the relationship between sleep efficiency and cardiovascular response to stress in a population with no identified pre-existing clinical conditions, minimizing the presence of potential clinical confounders.

It should be acknowledged that, while the current investigation used an ambulatory monitoring device, polysomnography is the gold standard for measuring sleep duration and efficiency. Actigraphy uses scoring algorithms that are based on the premise that the presence of movements indicates wakefulness and the absence of movements indicates sleep. This reliance of actigraphy on movement can cause errors in sleep measurement because actigraphy may over- or underestimate actual sleep. For example, if an individual is awake but motionless, it might be registered as “sleep” on the device. While the Actiwatch may sacrifice some accuracy in sleep measurement, it may improve generalizability by allowing individuals to sleep in their natural environments. Additionally, actigraphy is an economical methodology and has been validated against polysomnography in measuring sleep parameters^41^.

In summary, the current investigation used actigraphy to assess sleep efficiency and sleep duration in regularly exercising adults. We found that individuals with higher sleep efficiencies exhibited larger SBP and DBP recoveries after a cold pressor test, and demonstrated faster reaction times on a Stroop mental challenge test. These results suggest that sleep efficiency should be further investigated as a predictor for health outcomes, particularly in the context of cardiovascular disease. It is recommended that future research expand on the relationship between sleep efficiency, physiological response to stress, and acute or long-term cardiovascular outcomes, using polysomnography in an expanded sample size and over a longer period of time.
